# Topology Effect on Order–Disorder Transition of High-χ Block Copolymers

**DOI:** 10.1021/acs.macromol.4c00906

**Published:** 2024-07-18

**Authors:** Cheng-Yen Chang, Gkreti-Maria Manesi, Jiayu Xie, An-Chang Shi, Thanmayee Shastry, Apostolos Avgeropoulos, Rong-Ming Ho

**Affiliations:** † Department of Chemical Engineering, National Tsing Hua University, Hsinchu 30013, Taiwan, R.O.C.; ‡ Department of Materials Science Engineering, University of Ioannina, University Campus, Ioannina 45110, Greece; § Department of Physics and Astronomy, McMaster University, Hamilton, Ontario L8S 4M1, Canada

## Abstract

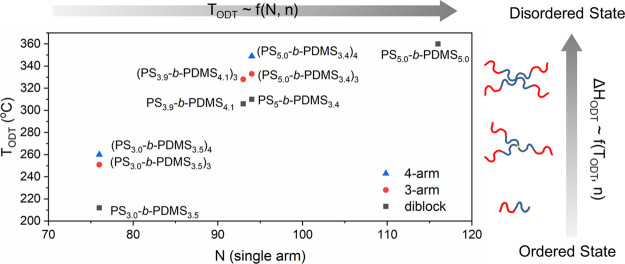

This work aims to systematically examine the topology effect on the self-assembly of block copolymers. Compositionally, symmetric polystyrene-*block*-polydimethylsiloxane block copolymers (BCPs) with different chain topologies (diblock, three-arm star-block, and four-arm star-block) and various molecular weights are synthesized. These purposely designed block copolymers are used as a model system to investigate the topology effect on order-to-disorder transition temperature (*T*_ODT_) by temperature-resolved small-angle X-ray scattering experiments. An increase of the *T*_ODT_ is observed when the arm number of BCPs with equivalent arm length (i.e., molecular weight) is increased from one to four. Based on the random-phase approximation (RPA), Flory–Huggins interaction parameter (χ) is determined from the regression of the measured *T*_ODT_. The observation by differential scanning calorimetry also demonstrates the shifting of the endothermic peak from the order-to-disorder transition of star-blocks to the higher temperature region, consistent with the scattering experiments and the RPA prediction.

## Introduction

The self-assembly of block copolymers (BCPs) has been extensively studied over the past few decades. A good understanding of the equilibrium phase behaviors, especially the AB-type block copolymers composed of two chemically distinct monomers (A and B), has been obtained.^1−5^ For linear AB diblock copolymers composed of A and B blocks covalently linked at their ends, the equilibrium phases are controlled by several parameters including the composition (*f*) of the copolymers and the block–block interaction strength quantified by the product χ*N* where χ represents the Flory–Huggins interaction parameter between A/B monomers and *N* is the degree of polymerization.^1,6−8^ Generally, the Flory–Huggins parameter χ is a decreasing function of the temperature *T*, usually assumed to have the form χ = A/*T* + B, where A and B are empirical constants.^9,10^ Due to the competition between A/B repulsion and chain connectivity, BCPs tend to form various ordered phases at low temperature.^11^ With the increase in the temperature, BCPs undergo an order-to-disorder transition (ODT) from an ordered phase to the homogeneous disordered phase. The phase diagrams of AB-type diblocks are commonly presented in the *f*-χ*N* plane. BCPs tend to form ordered phases when χ*N* is above a critical value (χ*N*_ODT_) that depends on the copolymer composition. Generally, the ODT of the copolymers assumes a parabolic-like curve that divides the ordered and disordered regions.^2,8^ For conformationally symmetric linear AB diblocks, the critical point is predicted to be at *f* = 0.5 and χ*N* = 10.495 by Leibler using the random-phase approximation (RPA).^1,2,4^ For asymmetric diblocks with *f* ≠ 0.5, the system undergoes a first-order ODT at a higher value of χ*N*. The specific value of χ*N*_ODT_ is a function of the A-block volume fraction *f*_A_, and it is determined by various factors such as conformational symmetry and copolymer architecture.^12−15^ For conformationally asymmetric linear AB-type diblocks, Matsen and Schick predicted that χ*N*_ODT_ as a function of *f* assumes an asymmetric shape and a higher critical value (χ*N*_ODT_ > 10.5).^16^ Hashimoto et al. explored the effect of molecular topology by a common PS-*b*-PI system sharing jointed junctions in the center as star-shaped architecture in the equilibrium state, discovering the remarkable dependence on the arm numbers of star-blocks to the *T*_ODT_s;^17,18^ the increase on arm numbers of PS-*b*-PI will increase the *T*_ODT_s and lower the χ*N*_ODT_. Further reports on the BCPs with different topologies (i.e., branched architecture) also revealed that the composition fluctuations and the non-Gaussian stretching in those blocks bonded at the jointed junctions can deeply alter the phase behaviors at a weak segregation limit.^19−22^ Specifically, BCP systems with nonuniform arms, for instance the miktoarm, possess a lower χ*N*_ODT_ such that ordered phases could be preserved at higher temperature or with lower *N*.^19,23^ Additional connections turning the molecular architectures into star-shaped topologies could improve the stability of the ordered equilibrium state. Apart from that, the newly emerged bottlebrush BCPs with polymer brushes grafted on the backbone also have a reduced χ*N*_ODT._^24−26^

Despite numerous studies on the ODT of various BCPs, the investigation of the phase behaviors of BCPs with high-χ has been rarely reported.^27−30^ The greatest advantage of such a system is that shorter chain lengths (*N*) are required for self-assembly as compared to low-χ BCPs.^31,32^ Theoretical predictions and experimental results have demonstrated that BCPs with a large χ value enable the formation of ordered structures with sharp compositional difference near the microphase-separated interface.^33−35^ Moreover, there are diverse and distinct phase behaviors discovered from self-assembled BCPs featuring high χ values, reflecting the effect of the strongly segregated interface.^36−38^ By taking advantage of the advances in synthetic chemistry, the creation of multiblock copolymers with various kinds of molecular topologies further expand the feasibility to unique, and meanwhile, significantly modified phase diagrams including the shifting on the ODT boundaries.^16,39^ Therefore, it is desirable to study the effect of chain topology on the phase behavior of the BCPs. A specific question is how would the topology effect alter the ODT?

In this study, polystyrene-*block*-polydimethylsiloxane (PS-*b*-PDMS) was selected as a representative system for the study of high-χ BCPs that is expected to have higher *T*_ODT_s. A series of compositionally symmetric PS-*b*-PDMS with various molecular weights and different topologies including three-arm and four-arm star-blocks were prepared for the study of the topology effect on the ODTs. In situ temperature-resolved small-angle X-ray scattering (SAXS) and thermal analysis by differential scanning calorimetry (DSC) were carried out to determine the *T*_ODT_s of the synthesized BCPs. Because the arms of the star-BCPs are identical PS-*b*-PDMS diblocks, the samples have the same composition. Therefore, any difference in the measured *T*_ODT_s should be attributed to the different topologies of the star-BCPs. Moreover, the increments of latent heat near ODT also reflects the topology effect on the first-order ODT of the system.

## Experimental Section

### Synthetic Procedure and Characterization of (PS-*b*-PDMS)_*n*_ (*n* = 1, 3, or 4)

Living diblock copolymer precursors (PS-*b*-PDMS)_*n*_ (*n* = 1) were synthesized by anionic polymerization through sequential addition of monomers under high vacuum conditions. Trimethylchlorosilane [(CH_3_)_3_SiCl] was used to terminate the reaction for the syntheses of the diblocks. Subsequently, the syntheses of the star-block copolymers (PS-*b*-PDMS)_*n*_ (*n* = 3 and 4) were accomplished by coupling the living diblock precursors with trichloromethylsilane (CH_3_SiCl_3_) and tetrachlorosilane (SiCl_4_), respectively. Detailed procedures for the syntheses of the diblock and star-block copolymers have been reported earlier by Ho and Avgeropoulos and co-workers (see Supporting Information for more details).^40^

### Sample Preparation

Cyclohexane (J.T. Baker, 99%) that is a neutral solvent for PS and PDMS was used to carried out solution casting of all bulk samples of (PS-*b*-PDMS)_*n*_ (*n* = 1, 3 or 4) at a concentration of 10 wt % at room temperature followed by thermal annealing at 100 °C for 3 days. Morphological observation by transmission electron microscopy (TEM) of the samples is provided in the Supporting Information.

### Morphological Observation

Ultrathin microsections (thickness less than 60 nm) of the solution-cast and thermally annealed (PS-*b*-PDMS)_*n*_ (*n* = 1, 3 or 4) were prepared at −160 °C by a Leica EM UC6 microtome and the accessories, Cryochamber EM FC7, for cryomicrotome. Real-space images (TEM) were acquired from the ultrathin microsections without staining due to the intrinsic mass contrast from PDMS to PS microdomains. TEM observations were performed on a JEOL-2100 microscope at an accelerating voltage of 200 kV.

### SAXS Experiments

The synchrotron beam source from beamline BL23A of the National Synchrotron Radiation Research Center was used for the SAXS experiments. A mirror monochromatic to the energy of 10 keV by a germanium (111) double-crystal monochromator was used to vertically focus the incident X-ray beam. The wavelength of the X-ray beam was 1.24 Å. The beam stop was a round tantalum disk 4 mm in diameter. A MAR CCD X-ray detector (MAR USA) was used to collect the two-dimensional (2D) SAXS patterns. Temperature-resolved SAXS experiments were carried out by step heating procedures from 30 °C to temperatures above the ODT for all synthesized samples. The corresponding SAXS profiles were recorded every 5 min.

## Results and Discussion

### Determination of *T*_ODT_ of PS-*b*-PDMS Diblock Copolymers

**Figure 1:**
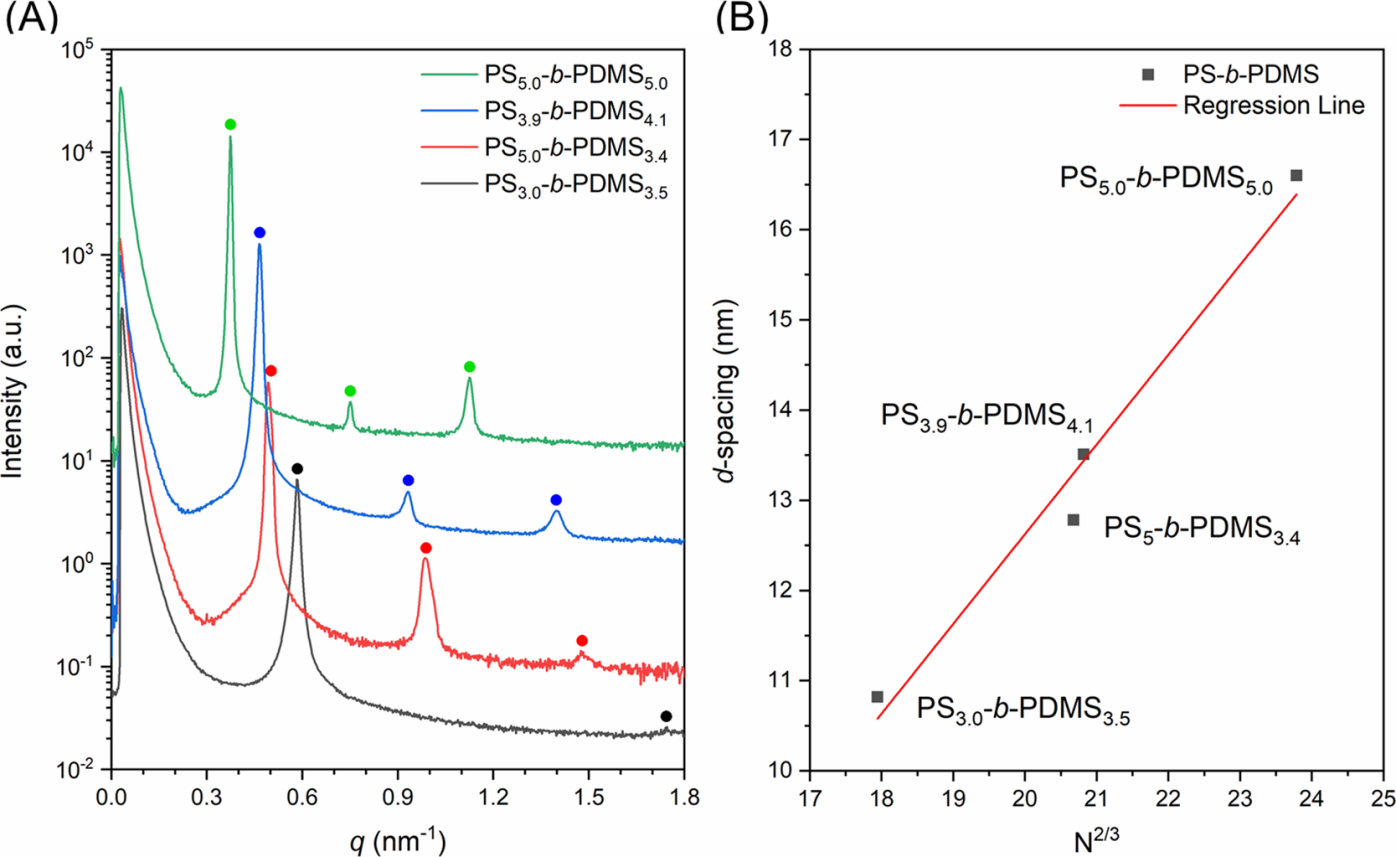
(A) One-dimensional SAXS profiles of PS-*b*-PDMS with different molecular weights. (B) *d*-spacing of PS-*b*-PDMS against degree of polymerization (*N*^2/3^) with linear fitting regression line according to the scaling relation (*d* ∼χ^1/6^*N*^2/3^).

**Figure 2:**
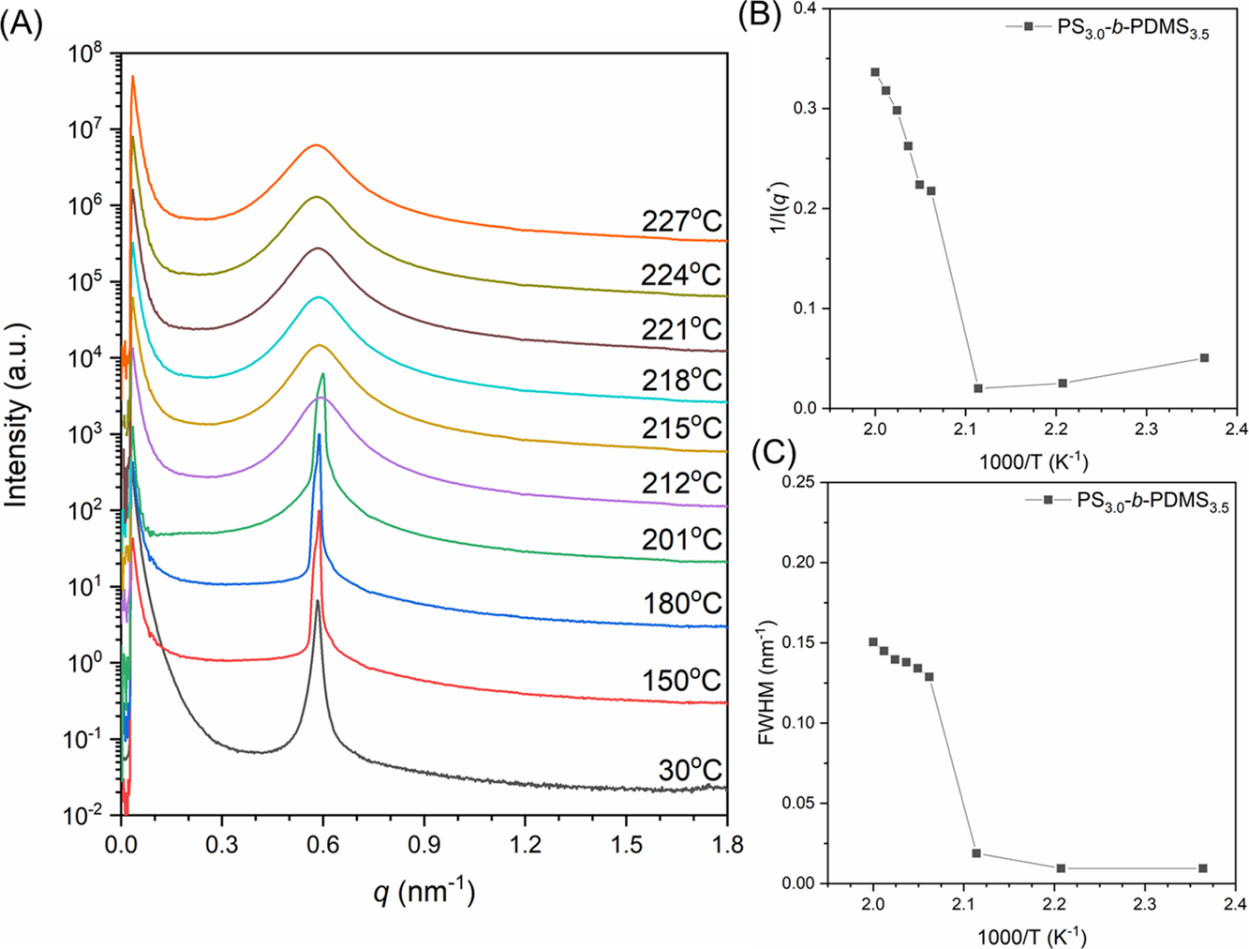
(A) In situ temperature-resolved one-dimensional SAXS profiles of PS_3.0_-*b*-PDMS_3.5_. Temperature dependence of (B) inverse of maximum intensity (1/*I*(q*)) and (C) fwhm derived from the left SAXS profile.

Figure 1A displays the SAXS profiles of four compositionally symmetric PS-*b*-PDMS diblocks with various molecular weights (see Figures S1 and S2 and Table 1 for detailed information). The scattering peaks at the relative *q* values of q/q* = 1:2:3 can be found from the samples, where the primary reflections are denoted as q*. The real-space images (Figure S3) of these samples exhibit lamellar morphologies. These observations confirm that the equilibrium phases of these four diblocks are all lamellae. The period or domain spacing (i.e., *d*-spacing) of the lamellae was obtained from the wavevector (q*) of the primary peak, *d* = 2π/ q*, resulting in a *d*-spacing of 10.8 12.8, 13.5, and 16.6 nm for these four samples. According to strong segregation theory, the period of the lamellae at the strong segregation limit follows the scaling relation of *d ∼* χ^1/6^*N*^2/3^. The observed period of the four samples is consistent with this scaling relation as shown in Figure 1B.^4^Figure 2A shows the results of an in situ SAXS experiment at which a step heating procedure was designed for the measurement of ODT temperature (*T*_ODT_) (see Experimental Section for details). Specifically, the ODT is signified by the disappearance of the sharp primary peak, the decrease of the peak intensity, and the broadening of the scattering function. Intense and sharp primary peaks observed at low temperatures become broadened and weakened as the temperature reaches 201 °C and eventually reach a smooth Gaussian-like curve at 212 °C. Furthermore, the intensities of the broadened peaks decrease as the temperature is increased. Figure 2B shows a plot of the inverse intensity of the primary peak [1/*I*(q*)] as a function of inverse temperature (1/*T*) based on the results shown in Figure 2A. The inverse intensity exhibits a slight decrease when the temperature is increased at low temperature, whereas it increases sharply at high temperature. The change of its behavior happens sharply at *T* ∼ 212 °C. This sudden jump of 1/*I*(q*) occurs at the same point where the sharp peaks from the ordered lamellae disappear, which could be identified as the ODT point. Consistent with these observations, a sharp transition could be identified from the plot of full width at half-maximum (fwhm) versus inverse temperature (1/*T*) that occurs at temperatures between 201 and 212 °C (Figure 2C). For PS_3.0_-*b*-PDMS_3.5_, the *T*_ODT_ of PS_3.0_-*b*-PDMS_3.5_ could therefore be determined at 212 °C. In comparison with the nonlinear change of 1/*I*(q*) versus 1/*T*, the steadily increase on fwhm when *T* > *T*_ODT_ makes it simpler to determine the transition point between the ordered and disordered states. As such, in what follows, we will use the changing point in the fwhm plots to determine the transition temperature *T*_ODT_, while the behavior of the 1/*I*(q*) plots will be used to confirm the results.

**Table 1:** Typical Characterization (Molecular Weights, Polydispersity, and Thermal Properties) of the Synthesized (PS-*b*-PDMS)_*n*_ (*n* = 1, 3 or 4)

sample	kg/mol^a^	kg/mol^a^	kg/mol^b^	*Đ*_M_^c^	d	(°C)^e^
PS_3.0_-*b-*PDMS_3.5_	3.0	3.5	6.5	1.05	0.59	64.7
(PS_3.0_-*b-*PDMS_3.5_)_3_	9.0	10.5	19.5	1.05	0.60	65.3
(PS_3.0_-*b-*PDMS_3.5_)_4_	12.0	14.0	26.0	1.06	0.60	65.8
PS_3.9_-*b-*PDMS_4.1_	3.9	4.1	8.0	1.07	0.52	65.3
(PS_3.9_-*b-*PDMS_4.1_)_3_	11.7	12.3	24.0	1.07	0.52	65.4
PS_5.0_-*b-*PDMS_3.4_	5.0	3.4	8.4	1.05	0.40	69.8
(PS_5.0_-*b-*PDMS_3.4_)_3_	15.0	10.2	25.2	1.06	0.40	70.2
(PS_5.0_-*b-*PDMS_3.4_)_4_	20.0	13.6	33.6	1.08	0.40	69.9
PS_5.0_-*b-*PDMS_5.0_	5.0	5.0	10.0	1.05	0.54	78.8

a Total number-average molecular weights corresponding to two individual blocks (PS and PDMS) determined by vapor pressure and membrane osmometry (VPO and MO).

b Total number-average molecular weight of the copolymers measured by VPO and MO.

c Polydispersity determined by size exclusion chromatography (SEC).

d Volume fraction of PDMS as calculated from proton nuclear magnetic resonance spectroscopy (^1^H NMR) using densities of PS and PDMS (ρ_PS_ = 1.04 g cm^–3^, ρ_PDMS_ = 0.97 g cm^–3^).

e Glass transition temperatures of both PS and PDMS segments determined by DSC.

**Figure 3:**
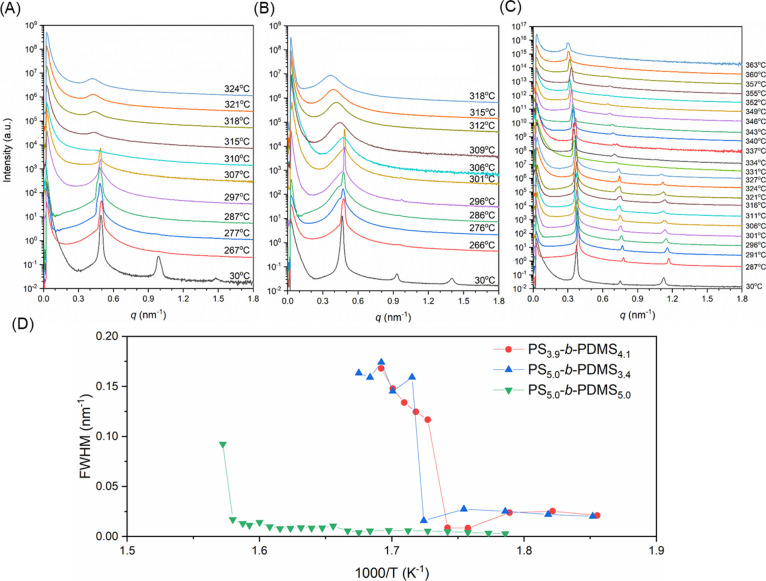
In situ temperature-resolved one-dimensional SAXS profiles of (A) PS_5.0_-*b*-PDMS_3.4_, (B) PS_3.9_-*b*-PDMS_4.1_, and (C) PS_5.0_-*b*-PDMS_5.0_. (D) Temperature dependence of fwhm derived from the corresponding SAXS profiles.

Figure 3A–C shows the in situ SAXS profiles of the other three diblocks, PS_5.0_-*b*-PDMS_3.4_, PS_3.9_-*b*-PDMS_4.1_, and PS_5.0_-*b*-PDMS_5.0_. Analogous ODT behaviors are observed in all cases, viz., the intensive primary reflection representing the ordered lamellae phase decays and becomes broaden as temperatures approach the transition point (*T*_ODT_). To accurately determine the transition point, the temperature dependence of the primary reflections in the SAXS profiles for the diblocks were analyzed in terms of fwhm as shown in Figure 3D; the transition temperatures (*T*_ODT_) determined from these fwhm plots were found to be 310, 306, and 363 °C. Similar steplike changes could be observed in the plot of 1/*I*(q*) as shown in Figure S4. These observations reveal that the ODT of the PS-*b*-PDMS diblock copolymers increases with the molecular weight of the samples, which is in agreement with the prediction from the SCFT.^2^

**Figure 4:**
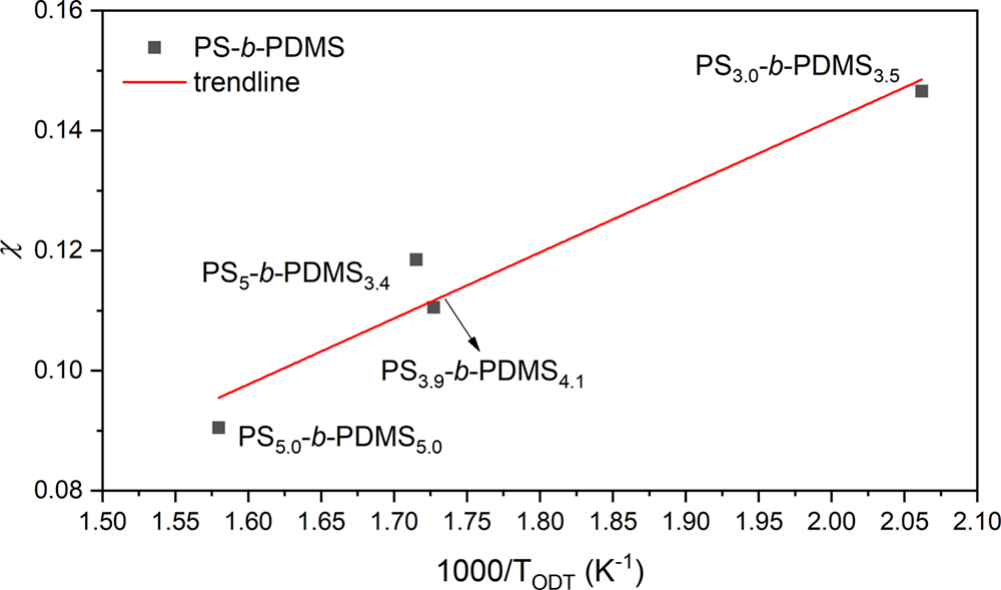
Temperature dependence of χ according to mean-field theory. The regression line (red) was fitted for calculation of χ as the function of temperature (χ = A/*T* + B).

The measured *T*_ODT_ from four diblocks with different molecular weights can be used to obtain the temperature dependence of χ. According to the mean-field theory, the critical segregation strength at ODT (χ*N*_ODT_) was calculated to be a constant regardless of the molecular weights when the composition is symmetric: χ*N*_ODT_ ∼ 10.495.^1,2,16^ The χ*N*_ODT_ due to the slight deviation from perfectly symmetric compositions of the four samples was calculated and used in the data analysis. The detailed characteristic information on the diblocks and star-blocks synthesized is summarized in Table 2. Accordingly, the χ – 1/*T* equation of the PS-*b*-PDMS can be estimated from the regression among the measured points of *T*_ODT_s from four diblocks as shown in Figure 4. It is found that the commonly used linear relationship χ(*T*) = A, *T*, B describes the data well. The parameters A and B were estimated from the linear regression:
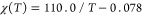


**Table 2:** Summarization of the Characteristics of (PS-*b*-PDMS)_*n*_ (*n* = 1, 3, 4) Synthesized and the Corresponding *T*_ODT_ Measured and (χ*N*)_ODT_ Based on the Calculation of RPA Prediction

sample	*N*^PS^	*N*^PDMS^	(χ*N*)_ODT_^a^	χ_ODT_	*T*_ODT_ (°C)^b^	q* (nm^–1^)^c^	*d*-spacing (nm)
PS_3.0_–*b*–PDMS_3.5_	29	47	11.14	0.147	212	0.581	10.8
(PS_3.0_–*b*–PDMS_3.5_)_3_	87	141	9.37	0.123	251	0.541	11.6
(PS_3.0_–*b*–PDMS_3.5_)_4_	116	188	7.57	0.100	260	0.543	11.6
PS_3.9_–*b*–PDMS_4.1_	38	55	10.50	0.111	306	0.465	13.5
(PS_3.9_–*b*–PDMS_4.1_)_3_	114	165	8.15	0.086	328	0.450	14.0
PS_5.0_–*b*–PDMS_3.4_	48	46	11.14	0.118	310	0.492	12.8
(PS_5.0_–*b*–PDMS_3.4_)_3_	144	138	8.18	0.090	333	0.487	12.9
(PS_5.0_–*b*–PDMS_3.4_)_4_	192	184	7.53	0.080	349	0.487	12.9
PS_5.0_–*b*–PDMS_5.0_	48	68	10.50	0.090	363	0.379	16.6

a Critical segregation strength at ODT obtained from the RPA calculation.

b Measured *T*_ODT_ of the synthesized (PS-*b*-PDMS)_*n*_ (*n* = 1, 3, 4) by in situ SAXS.

c Positions of the primary peaks of the (PS-*b*-PDMS)_*n*_ (*n* = 1, 3, 4) by SAXS.

Using this equation, the χ value of PS-*b*-PDMS at 100 °C is estimated to be 0.22, consistent with the predicted values acquired from the previous report.^41^

### ODT of Star-Block (PS-*b*-PDMS)_*n*_

**Figure 5:**
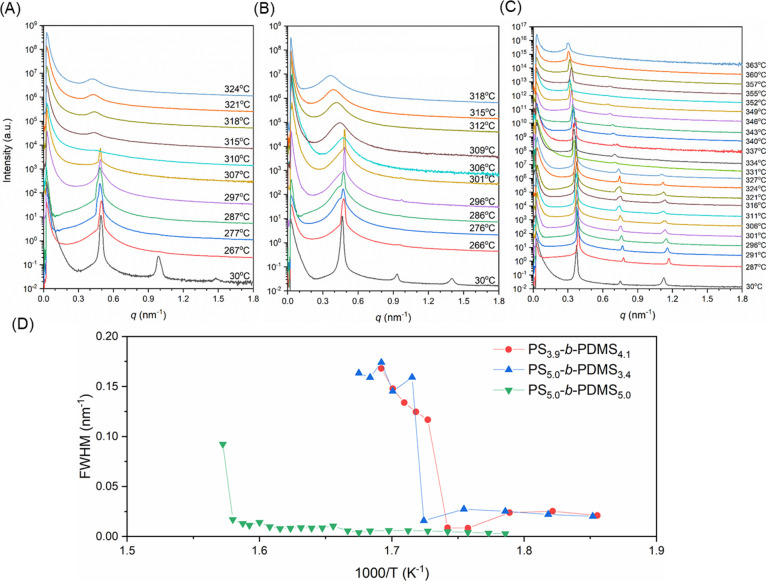
In situ temperature-resolved one-dimensional SAXS profiles of (A) (PS_3.0_-*b*-PDMS_3.5_)_3_, (B) (PS_3.9_-*b*-PDMS_4.1_)_3_, and (C) (PS_5.0_-*b*-PDMS_3.4_)_3_. (D) Temperature dependence 251 °C of fwhm derived from the corresponding SAXS profiles.

Here, we examine the ODT of the lamellae-forming star-block (PS-*b*-PDMS)_3_ with the same arm length (Table 1). Figure 5 shows results obtained from in situ SAXS experiments for three-arm star-blocks, (PS_3.0_-*b*-PDMS_3.5_)_3_, (PS_3.9_-*b*-PDMS_4.1_)_3_, and (PS_5.0_-*b*-PDMS_3.4_)_3_, at temperatures from 178 to 339 °C following the same heating procedures as that for the diblocks. As shown in Figure 5A, the second-order reflection of self-assembled (PS_3.0_-*b*-PDMS_3.5_)_3_ vanished at 227 °C; the primary reflection gradually decreases when the temperature is increased up to 251 °C. A sharp transition can be clearly identified in the 1/*I*(q*) plot as shown in Figure S5. Similar discontinuity in the fwhm plot against temperature is also observed between 247 and 247 °C (Figure 5D). In comparison with the diblock counterpart PS_3.0_-*b*-PDMS_3.5_, the observed *T*_ODT_ is risen over 30 °C. The vanishing points of the primary reflections of the other self-assembled three-arm star-blocks are also shifted to higher temperatures as shown in Figure 5B,C. The second-order reflections can still be identified at the high-temperature region, implying a higher degree of ordering as compared to the diblocks. Moreover, the corresponding *d*-spacing remains similar to diblocks (see Table 2). For (PS_3.9_-*b*-PDMS_4.1_)_3_ and (PS_5.0_-*b*-PDMS_3.4_)_3_, the step changes on the 1/*I*(q*) (Figure S5) and fwhm (Figure 5D) were identified at 328 and 333 °C, respectively. Interestingly, a significant increment in *T*_ODT_ is found in all three-arm star-blocks, implying that the ODT of the BCPs is greatly affected by altering the molecular topology.

**Figure 6:**
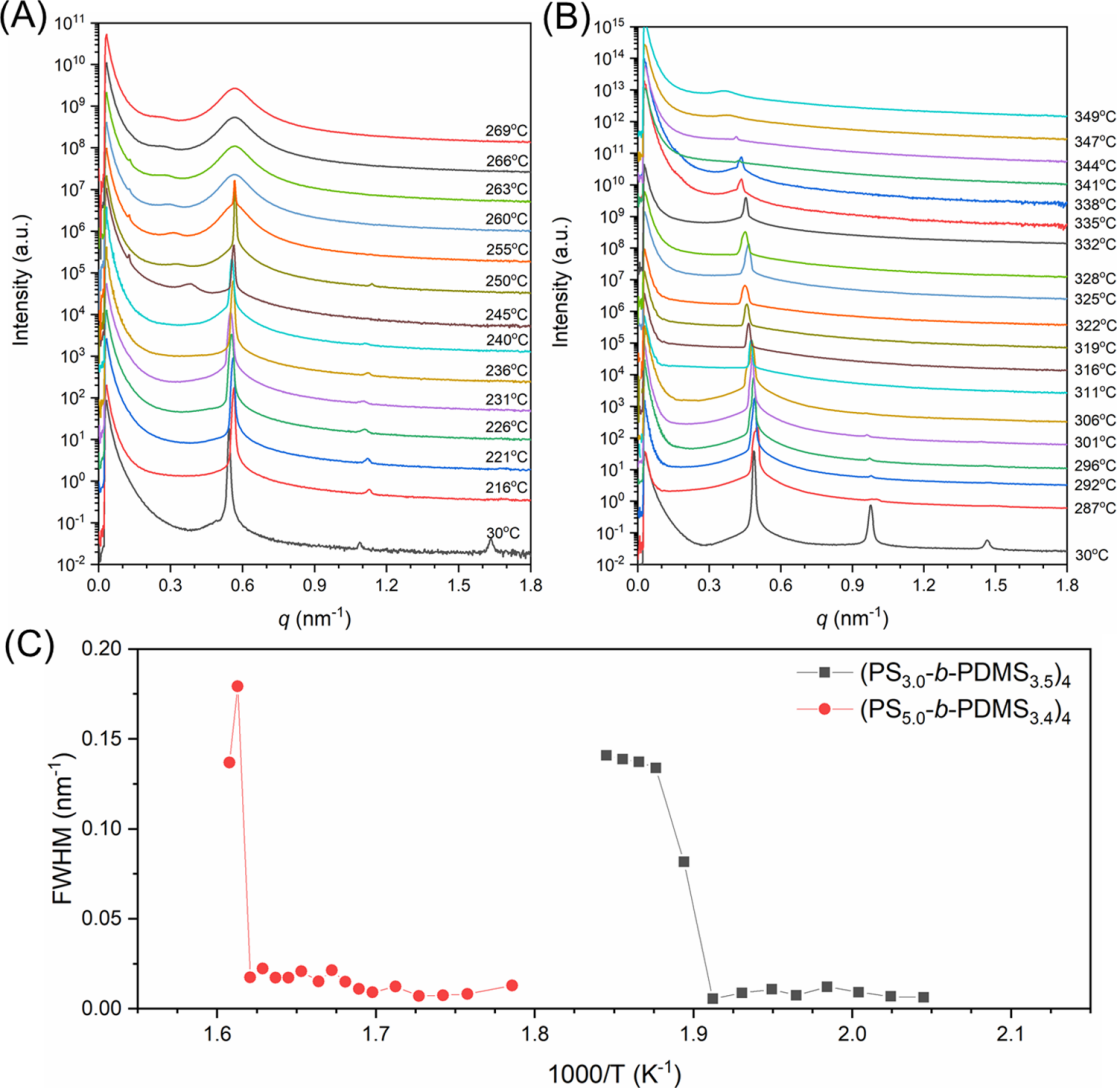
In situ temperature-resolved SAXS one-dimensional SAXS profiles of (A) (PS_3.0_-*b*-PDMS_3.5_)_4_, and (B) (PS_5.0_-*b*-PDMS_3.4_)_4_. (C) Temperature dependence of fwhm derived from the corresponding SAXS profiles.

**Figure 7:**
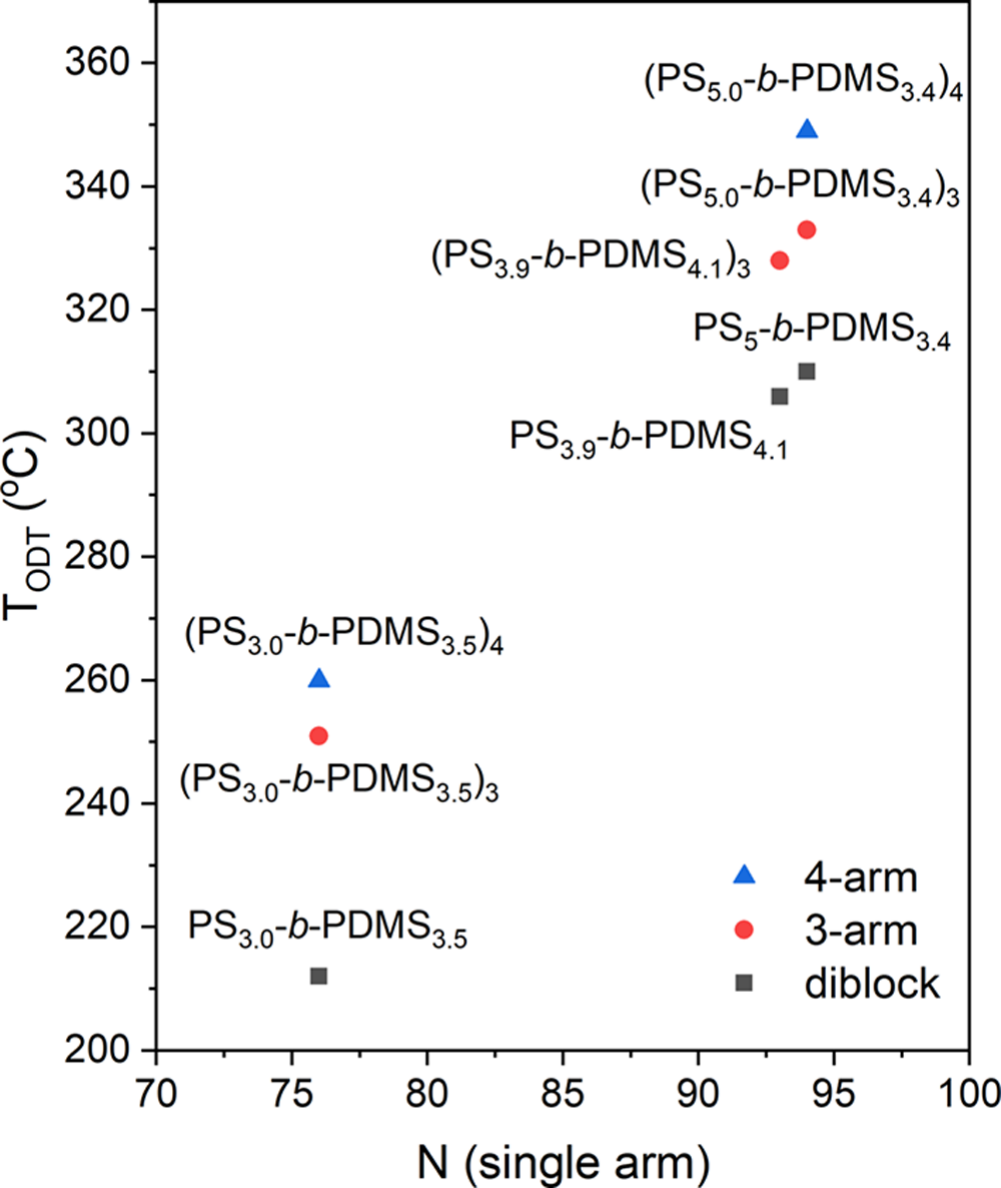
Plot of ODT temperatures (*T*_ODT_) of (PS-*b*-PDMS)_*n*_ (*n* = 1, 3, and 4) against the length of the diblock arms in (PS-*b*-PDMS)_*n*_ (*n* = 1, 3, and 4) and the numbers of diblock arms.

Two four-arm star-block (PS-*b*-PDMS)_4_ with the same arm length as their diblock precursors and three-arm star-blocks were synthesized for further investigation of the arm number effect on the ODTs of BCPs (see Table 2 for details). A clear ODT of (PS_3.0_-*b*-PDMS_3.5_)_4_ can be identified at temperatures between 255 and 260 °C as shown in Figure 6A. The temperature-dependent 1/*I*(q*) (Figure S6) and fwhm (Figure 6C) are plotted based on the corresponding SAXS profile where the discontinuity in both terms occurred at 260 °C. Similarly, the sharp transition could be found in (PS_5.0_-*b*-PDMS_3.4_)_4_ (Figure 6B,C); moreover, the longer arm length as compared to (PS_3.0_-*b*-PDMS_3.5_)_4_ results in higher *T*_ODT_. In particular, the *T*_ODT_s for the four-arm star-blocks are always higher than those of three-arm star-blocks as well as diblocks. Figure 7 shows a summary of the measured *T*_ODT_s for all samples examined in the current study. Two obvious trends can be identified from the results shown in Figure 7. (1) The ODT temperature *T*_ODT_ increases when the arm length increases, which is consistent with the prediction from the self-consistent field theory.^16,39^ (2) Higher *T*_ODT_ is observed when the number of diblock arms is increased, demonstrating that the topology of the star-block copolymers affects the ODT significantly.

### Topology Effect on ODT

**Figure 8:**
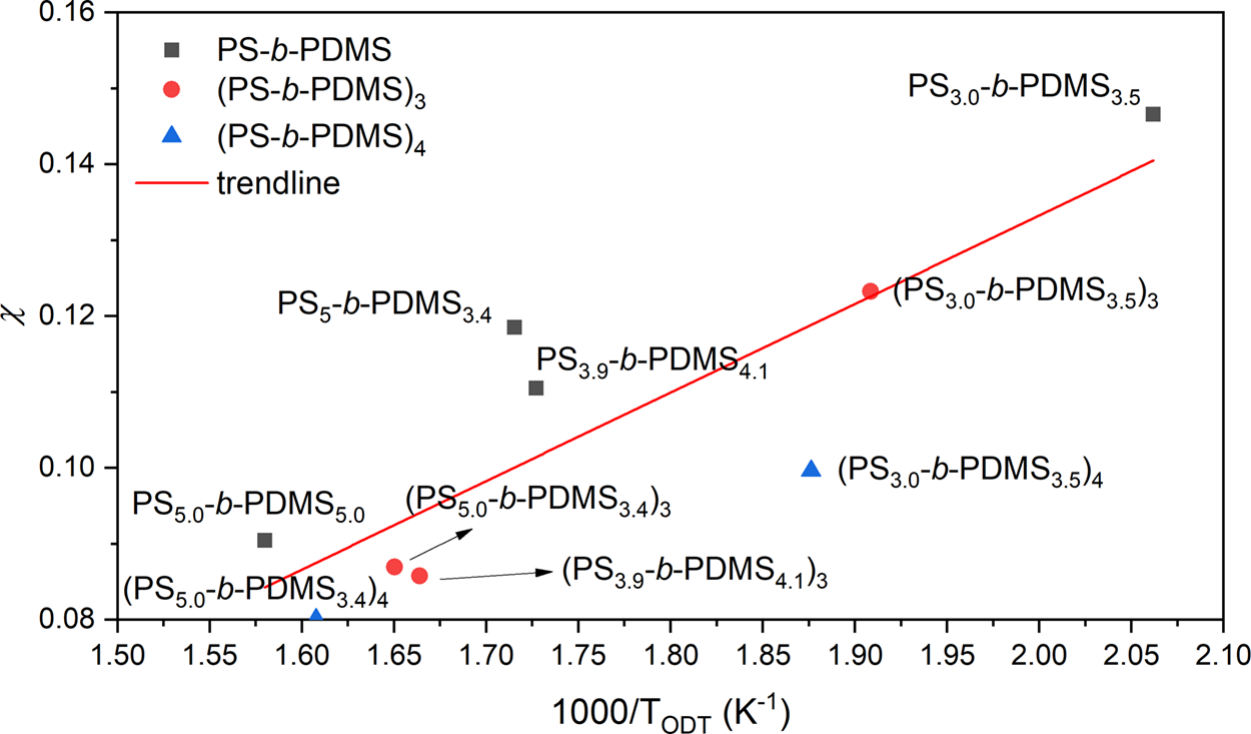
Temperature-dependent χ value plotted against measured *T*_ODT_. Trendline was derived from the experimental points in the plot. Mean-field theory is applied to the assumption of critical segregation strength of diblocks and three-arm and four-arm star-blocks.

In the past decades, several reports appeared to survey the thermodynamics of the BCPs with multiarm topology.^17,18,39,42^ A common conclusion from those reports is that the degree of polymerization (*N*) of the star-blocks can be represented by the *N* from one of the identical diblock arms because of the nearly invariant *d*-spacing in self-assembled melts. Table 2 summarizes the measured *T*_ODT_ of all synthesized (PS-*b*-PDMS)_*n*_ (*n* = 1, 3, and 4), which are used in the linear regression of χ against 1/*T*. The χ*N*_ODT_ for a diblock and star-blocks were obtained by RPA calculations, showing that χ*N*_ODT_ decreases as the arm number increases. Detailed descriptions of the RPA calculation are provided below and in the Supporting Information. As shown in Figure 8, the linear equation for χ – 1/*T* for diblocks and star-blocks are consistent because of the fact that the interaction parameter describes the PS to PDMS interactions and thus should be insensitive to the chain topology.^2,16,39^ Notably, the slight conformational asymmetry (α = *b*_A_^2^/*b*_B_^2^) of PS-*b*-PDMS (α ∼ 1.5) might result in the calculated χ.^16,43,44^ Overall, the calculated (χ*N*)_ODT_ for the diblocks and star-blocks examined in this study by RPA show reasonable consistency within the high-temperature region. Because of the expected enthalpic contribution from the same repulsive interaction between the constituted components, a linearly empirical equation for the χ – 1/*T* relationship of PS-*b*-PDMS can be estimated from the measured points from the diblocks and three-arm and four-arm star-blocks:
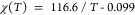


To investigate the *T*_ODT_ from the diblocks and star-blocks, we calculated the spinodal line for these systems by using the RPA applied to a simplified model of incompressible diblocks and star-blocks modeled as continuous Gaussian chains. For the model of star-BCPs with *n* arms, their total degree of polymerization (*N*_t_) *N*_t_ = *n*(*N*_A_ + *N*_B_) = *nN*. The collective structural factor (*S̃*) of the self-assembled PS-*b*-PDMS can be calculated following the standard procedure.^1,45^ The two-component density–density correlation function defined by RPA is given below (eqs 1 and 2):
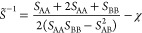
1where
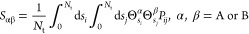
2Here, *S* denotes the correlation function between the constituted monomers (i.e., monomers of PS and PDMS in this case). For the incompressible melt of star-blocks, we assume that each AB-type star-block copolymer (BA)_*n*_ chain is composed of *n* identical arms covalently bonded at their A (i.e., PDMS) ends, and all of the B (i.e., PS) ends are free. With these assumptions, the integrals of *S*_αβ_ to get the expressions for the various *S*_αβ_’s can be carried out using eqs 3to 5:
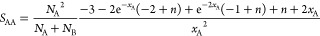
3
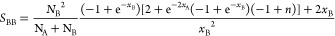
4
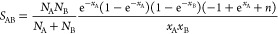
5where
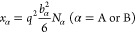


**Figure 9:**
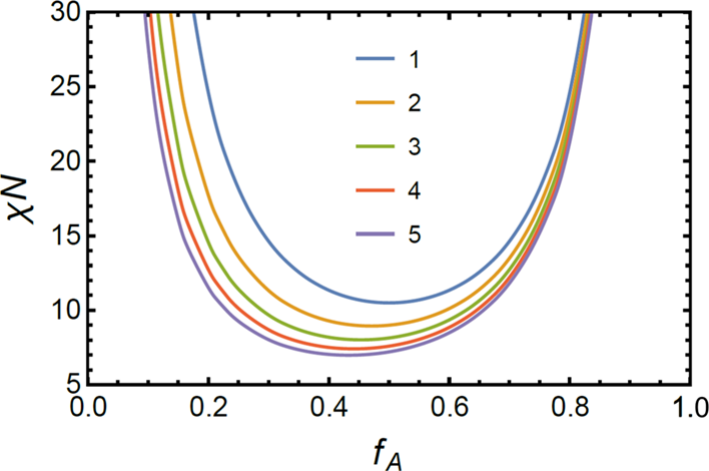
Theoretical phase diagram based on RPA for self-assembled symmetric-composition PS-*b*-PDMS with different topologies including diblocks (blue), triblock (orange), and three-arm (green), four-arm (red), and five-arm star-blocks (purple). Ordered phases are located at interior regions, while fluctuated state with disorder structures are out of the curves.

The criterion for the ODT is that the minimum of *S̃*^–1^ goes to zero (Figure S7). For symmetric diblocks, the minimum value of *S̃*^–1^ reaches 0 when χ*N* = 10.495. As shown in Figures 9 and S8, an increase of the number of diblock arms leads to a lower value of (χ*N*)_spinodal_; in other words, a higher *T*_ODT_ is expected, consistent with previous SCFT prediction^16^ and our experimental observations.

**Figure 10:**
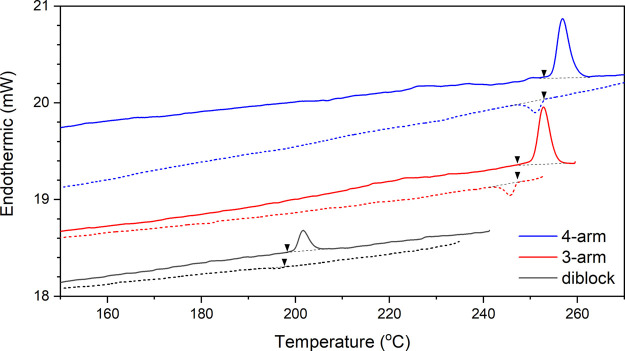
DSC thermograms of (PS_3.0_-*b*-PDMS_3.5_)_*n*_ (*n* = 1, 3, and 4) synthesized. The measurements of the latent heat at the ODTs (Δ*H*_ODT_) were calculated from the first cycle during measurements at the heating and cooling rates of 20 °C/min.

As reported, the variation of molecular topology would modify the phase behaviors of BCPs.^39,46,47^ Specifically, the self-assembled BCPs with designed branched topologies may result in higher energy barriers to transform the self-assembled ordered states into a fluctuated state. BCPs with more complex topologies could exhibit higher *T*_ODT_s. This effect could be explored by examining the latent heat at ODTs (Δ*H*_ODT_), which may reflect the degree of segregation strength (χ*N*) as well as the ordering^48^Figure 10 displays the DSC thermogram of the family of (PS_3.0_-*b*-PDMS_3.5_)_*n*_ (*n* = 1, 3, and 4). The heating and cooling rates for scanning of the self-assembled PS-*b*-PDMS were fixed at 20 °C/min from 150 to 270 °C. The endothermic peaks during the first heating cycle indicate that a first-order transition takes place near ODTs.^48−50^ The four-arm star-block gives the highest *T*_ODT_ followed by the three-arm star-block and, last, the diblock, consistent with the in situ SAXS experiments. Moreover, the reversible ODTs show exothermic peaks after cooling from the fluctuated state; the kinetics of rearrangements of polymer chains, however, result in lower latent heat than that acquired from the heating cycle. The measured normalized Δ*H*_ODT_ (mW/g) from heating cycles for diblock, and three-arm and four-arm star-blocks are 0.22, 0.57, and 0.68 mW/g, respectively. Because the diblock and star-block samples have the same arm lengths, the total molecular weights of star-blocks are exactly *n* times that of one single arm (i.e., diblock); in other words, there will be fixed numbers of “diblocks” at a given weight regardless of the topology of star-blocks. Interestingly, the normalized Δ*H*_ODT_ value of the three-arm star-block is over twice that of the diblock; the four-arm star-block has the highest value, approximately 3 times that of the diblock. According to the mean-field theory, the possible maximum enthalpy at ODTs can be estimated by the following equation: Δ*H*_max_ ∼ *RTf*_A_*f*_B_(χ*N*)_ODT_/*M*_*n*_.^48^ However, the increment of the enthalpy may not originated from the possible increase on (χ*N*)_ODT_ where the four-arm star-block shows the lowest value (χ*N*_ODT_ ∼ 8); the diblock instead exhibits the highest segregation strength (χ*N*_ODT_ = 10.495) at ODTs. The great changes in latent heat during the ODTs include the individual contributions from both enthalpic and entropic changes from two components while breaking the segregated interface. Intuitively, the major difference will be from the entropic term due to the consistent polymer–polymer interface (i.e., similar enthalpic change). A simple approximation based on the ideal solution model (Δ*H*_ODT_ = *T*_ODT_ × Δ*S*_ODT_) might reflect the variation not only from the change of *T*_ODT_ but also the entropic change. More significant deviations on entropic changes among the diblock and star-blocks can be observed during the first cooling cycle when the ordered lamellae phase recovers from the disordered state near the *T*_ODT_ (Figure S9 and Table S1). Accordingly, those results imply that the self-assembled star-blocks will show larger entropic variation than the diblocks; hypothetically, geometric confinement from the jointed junctions in star-blocks due to the bridging conformation in the self-assembled morphologies could be significant on the transition from ordered to disordered state.^40,51^ For linear BCPs, the polymer chains will be actually stretched to reach the minimum energy state near the ODT as the clustering-like monomers become “polarized.”^52,53^ However, a “dumbbell” shape was found in the miktoarms rather than random Gaussian coils.^54^ Similarly, the non-Gaussian stretching will also occur near the ODT for star-blocks. As a result, the “crowding” effect in star-blocks will constrain the chain packings and therefore result in a large variation on endothermic variation at the ODT of self-assembled star-blocks.

## Conclusions

In summary, the topology effect on the ODT of BCPs was examined by in situ temperature-resolved SAXS and thermal analysis by DSC. A series of diblock and star-block PS-*b*-PDMS with nearly symmetric compositions were synthesized for systematic investigation. It is observed that the transition points (*T*_ODT_) of the star-blocks are significantly higher than those of the diblocks; *T*_ODT_s for four-arm star-blocks are higher than those for the three-arm star-blocks. Moreover, the periods or domain spacings of the star-blocks remain the same as their diblock precursors with the same arm lengths. The variation of the conformational topology mainly affects the ODT instead of the dimensions of the self-assembled structure. The prediction by RPA further validates the reduction in χ*N*_ODT_ as the arm number of BCP increases, at which the χ*N*_ODT_ of a star-block is reduced lower than that (χ*N*_ODT_ ∼ 10.495) for a diblock. By fitting the measured *T*_ODT_s for all samples, we obtained an empirical linear relationship of χ against 1/*T*. The observation by DSC also shows a shifting of the endothermic peaks for the ODT to higher temperature with the increase of the arm number of the BCP, consistent with the in situ SAXS results. Furthermore, there are significant discrepancies in the latent heats among the self-assembled diblocks and star-blocks near the ODT. A simple approximation to the first-order transition reveals that the self-assembled star-blocks would show larger entropic variation than the diblocks, implying that the geometric confinement on molecular conformation may lead to crowding effect on the chain packing in self-assembled star-blocks. Accordingly, the topology effect on the ODT will lead to collective variation, including a decrease in the χ*N*_ODT_ for microphase-separated interfaces and an increase in the entropic contribution due to the bonded PDMS chain ends at the star-shaped junction.

## Supporting Information Available

Detailed descriptions for synthesis of (PS-*b*-PDMS)_*n*_ (*n* = 1, 3, 4); details of the in situ SAXS experiments and the analyses of SAXS results; setups for prediction of ODTs by RPA; and details of DSC thermograms of self-assembled (PS-*b*-PDMS)_*n*_ (*n* = 1, 3, 4) near ODT (PDF)
