# Supplementary material for: Topology Effect on Order–Disorder Transition of High-χ Block Copolymers
Source: Macromolecules. 2024 Jul 18;57(15):7087–97. doi: 10.1021/acs.macromol.4c00906 (PMC11325650; doi:10.1021/acs.macromol.4c00906)
Supplement: Supplementary file 1 — ma4c00906_si_001.pdf [file ma4c00906_si_001.pdf]

Supporting Information for

Topology Effect on Order-Disorder Transition of High-χ Block Copolymers

Cheng-Yen Chang,[†,‡] *Gkreti-Maria Manesi,*[¥,‡] *Jiayu Xie,*[#,‡] *An-Chang Shi,*[*, #] *Thanmayee Shastry,*[†] *Apostolos Avgeropoulos,*[*, ¥] *and Rong-Ming Ho*[*,†]

[†] Department of Chemical Engineering, National Tsing Hua University, Hsinchu 30013, Taiwan, R.O.C.

[¥] Department of Materials Science Engineering, University of Ioannina, University Campus, Ioannina 45110, Greece

[#] Department of Physics and Astronomy, McMaster University, Hamilton, Ontario L8S 4M1, Canada

[‡] C.-Y. Chang, G.-M. Manesi and J. Xie contributed equally.

* To whom correspondence should be addressed.
Tel: 1- 905-525-9140; E-mail: shi@mcmaster.ca
Department of Physics and Astronomy, McMaster University, Hamilton, Ontario L8S 4M1, Canada

Tel: 30-26510-09001; Fax: 30-26510-07030; E-mail: aavger@uoi.gr
Department of Materials Science Engineering, University of Ioannina, University Campus, Ioannina 45110, Greece

Tel: 886-3-5738349; Fax: 886-3-5715408; E-mail: rmho@mx.nthu.edu.tw
Department of Chemical Engineering, National Tsing Hua University, Hsinchu 30013, Taiwan, R.O.C.

Table of Contents

Synthesis of Linear Diblock and Star-Block Copolymers of (PS-*b*-PDMS)$_n$ (n = 1, 3, 4). 2

Sample Preparation 5

Morphological Observation 5

In-situ Temperature-Resolved Small-Angle X-ray Scattering (SAXS) Experiments. 6

Setups for Prediction of ODTs by Random Phase Approximation (RPA) 8

Measurements of Latent Heat of Self-Assembled (PS-*b*-PDMS)$_n$ at ODTs 11

Synthesis of Linear Diblock and Star-Block Copolymers of (PS-*b*-PDMS)$_n$ (n = 1, 3, 4).

Styrene (Acros Organics, 99%), hexamethylcyclotrisiloxane (D_3, Sigma-Aldrich, 98%), secondary butyllithium (*sec*-Buli, Sigma-Aldrich, 1.4 M in cyclohexane), benzene (Chem-Lab, 99.7%) and tetrahydrofuran (THF, Carlo Erba, 99.9%) were meticulously purified to meet the high standards for anionic polymerization to synthesize polystyrene-*block*-polydimethoxysilane (PS-*b*-PDMS).[1] Following the established techniques for synthesis of PS-*b*-PDMS,[1] the diblock (PS-*b*-PDMS)$_n$ (n = 1) was synthesized by utilizing styrene as a monomer (10 g, 0.096 mol) and *sec*-Buli as an initiator (2.40 mmol) in a non-polar environment (benzene 400 ml) and the reaction proceeded for 18 hours. After retrieving a small aliquot to trace the degreee of polymerization by size exclusion chromotagraphy (SEC) as shown in **Figure S1** and vapor pressure osmometry (VPO), D_3 (13 g, 0.17 mol) was introduced in the reacting flask and remained for another 18 hours in order to accomplish the ring opening of D_3. Equal quantity of THF (400 ml) was added to the solution in order to propagate the polymerization of D_3 for 4 hours at ambient conditions and then quenched to -20℃ holding for 10 days. After 10 days, the solution was divided into three separate flasks. Chlorotrimethylsilane ($(CH_3)_3SiCl$, Sigma-Aldrich, 98%) was purified in advance and added in one of the flasks to terminate the reaciton. In the rest two flasks, coupling agents were stoichiometrically introduced to carry out the preparation of three-arm and four-arm star-block (PS-*b*-PDMS)$_n$ (n = 3, 4) based on the same source of the diblock precursor. In the case of three-arm star-block (PS-*b*-PDMS)$_n$ (n = 3), purified trichloromethylsilane (CH_3SiCl_3, Sigma-Aldrich, 98%) (0.4 mmol) was added to the solution to initiate the coupling reaction which lasted for approximately 1 month prior to termination with $(CH_3)_3SiCl$. Same protocol was adopted for the synthesis of the four-arm star-block (PS-*b*-PDMS)$_n$ (n = 4) by exploiting purified tetrachlorosilane ($SiCl_4$, Sigma-Aldrich, 99%) (0.3 mmol) as the coupling reagent. After termination, all synthesized materials were precipitated in cold methanol and

subsequently transferred into a vacuum oven to remove residual solvents. In contrast to diblock, star-block PS-*b*-PDMS were further submitted to fractionation to remove all the by-products including homopolymers and precursor. Detailed procedures for the syntheses of the diblock and star-block copolymers from a diblock precursor have been reported by Ho and Avgeropoulos *et al.*[1] Eventually, the narrow-dispersed $(PS\text{-}b\text{-}PDMS)_n$ (n = 1, 3, 4) were prepared as evidenced by the SEC (**Figure S1** and **Table 1**). Extensive characterizations by ^{1}H-NMR (**Figure S2**) and differential scanning calorimetry were carried out to determine the volume fraction and its thermal properties (**Table 1**).

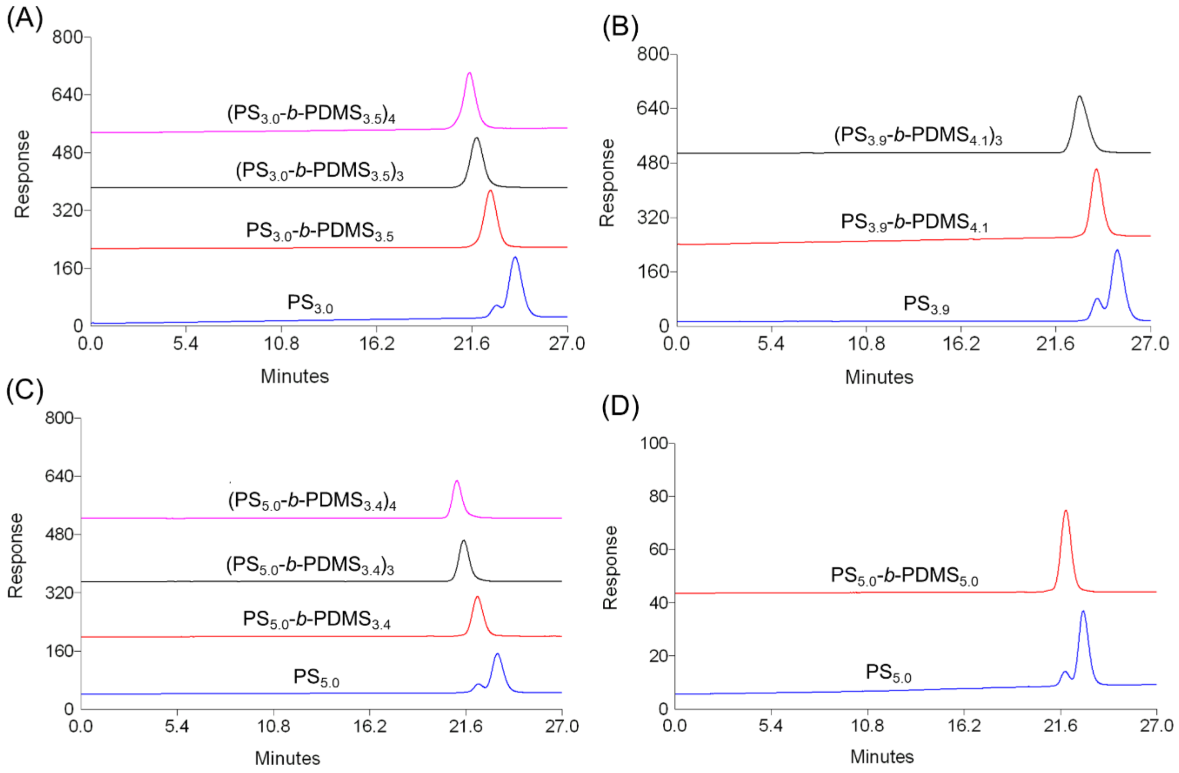

Figure S1. Chromatographs of the synthesized $(PS\text{-}b\text{-}PDMS)_n$ (n = 1, 3 or 4): (A) $PS_{3.0}$-*b*-$PDMS_{3.5}$, (B) $PS_{3.9}$-*b*-$PDMS_{4.1}$, (C) $PS_{5.0}$-*b*-$PDMS_{3.4}$ and (D) $PS_{5.0}$-*b*-$PDMS_{5.0}$. The results of PS precursors were presented in blue; the SEC curves of diblocks (red), three-arm (black) and four-arm star-blocks (pink) are arbitrarily shifted and stacked on each chromatograph.

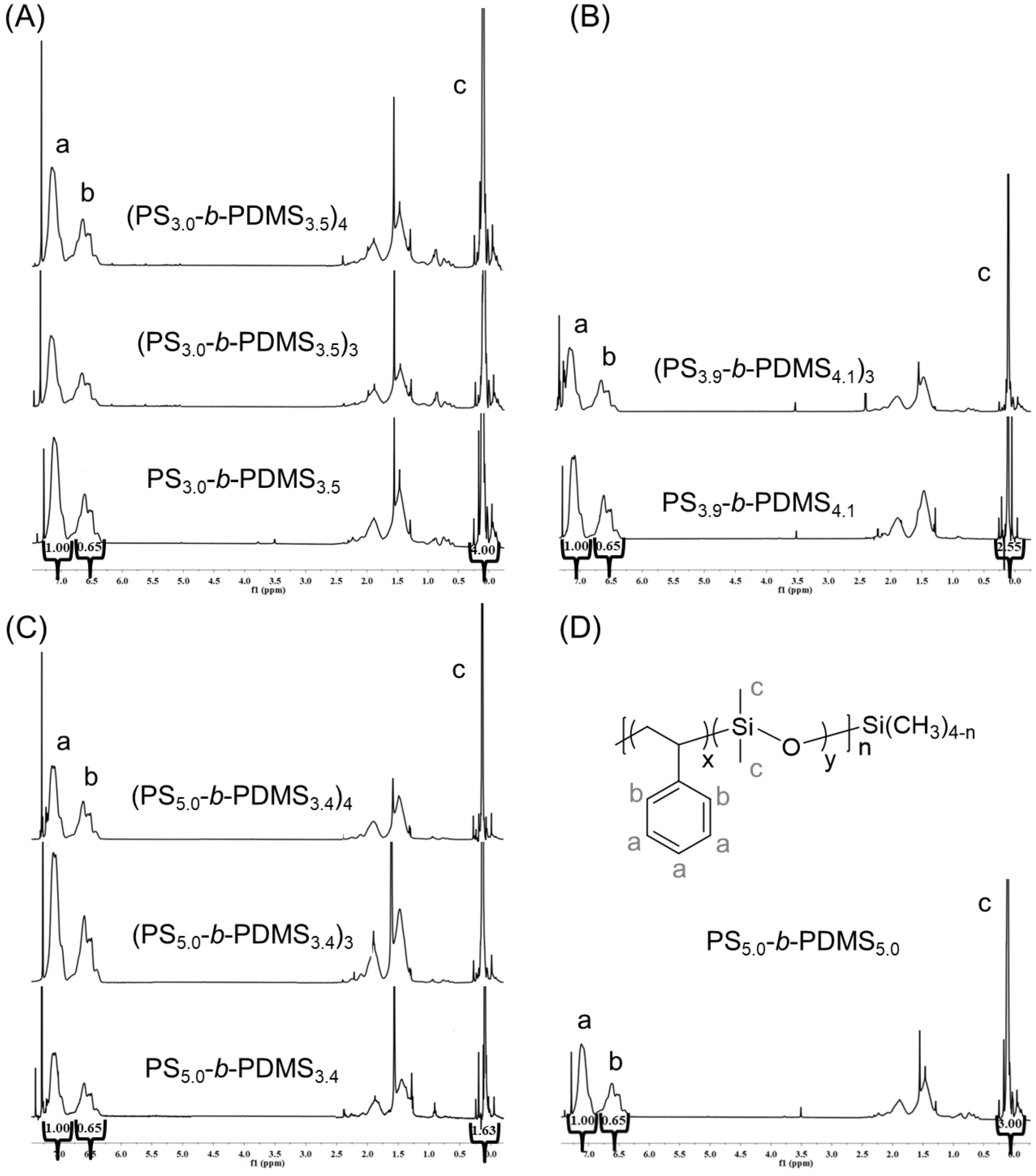

Figure S2. ^{1}H-NMR spectra (in $CDCl_3$) of the synthesized (PS-*b*-PDMS)$_n$ (n = 1, 3 or 4). (A) $PS_{3.0}$-*b*-$PDMS_{3.5}$, (B) $PS_{3.9}$-*b*-$PDMS_{4.1}$, (C) $PS_{5.0}$-*b*-$PDMS_{3.4}$ and (D) $PS_{5.0}$-*b*-$PDMS_{5.0}$. The chemical structure of (PS-*b*-PDMS)$_n$ is provided in the inlet. The ratios of peak integrals to the aromatic ring (a and b, 6.3 – 7.5 ppm) in PS blocks and to the methyl groups (c, ~ 0 ppm) in PDMS blocks are calculated for determination of volume fraction (f_{PDMS}^{v}).

Sample Preparation

All bulk samples of (PS-*b*-PDMS)$_n$ (n = 1, 3, 4) were prepared by the solution casting at ambient condition using cyclohexane (C_6H_{12}) which can be referred as a neutral solvent for both PS and PDMS segments. The concentration of polymer solution was fixed at 10 wt %. Slow evaporation rate of solvent (< 0.1 ml/day) was applied to avoid of the formation of kinetically trapped morphologies. Subsequently, all bulk samples were detached from glass vials and then transferred to a vacuum oven for 1 day for removal of residual solvent. The as-cast samples were sealed in quartz tubes under vacuum for the following thermal annealing at 100℃ for three days.

Morphological Observation

Ultrathin microsections (thickness lower than 60 nm) of the solution-cast and the thermally annealed (PS-*b*-PDMS)$_n$ (n = 1, 3 or 4) were prepared at -160□ by a Leica EM UC6 microtome with accessory for cryo-microtome (Cryochamber EM FC7). Real-spacing images (TEM) were acquired from the ultrathin microsections without staining due to intrinsic mass-thickness contrast from PDMS to PS microdomains. TEM studies were performed on a JEOL-2100 Transmission electron microscopy (TEM) operating at an accelerating voltage of 200 kV.

For further examination of the intrinsic phase behaviors of (PS-*b*-PDMS)$_n$ (n = 1, 3 or 4) synthesized, four diblocks were prepared for real-space observation under TEM. As shown in **Figure S3**, the alternating dark and bright stripes evidenced the formation of lamellar morphology, well consistent with the obtained scattering profiles shown in **Figure 1**.

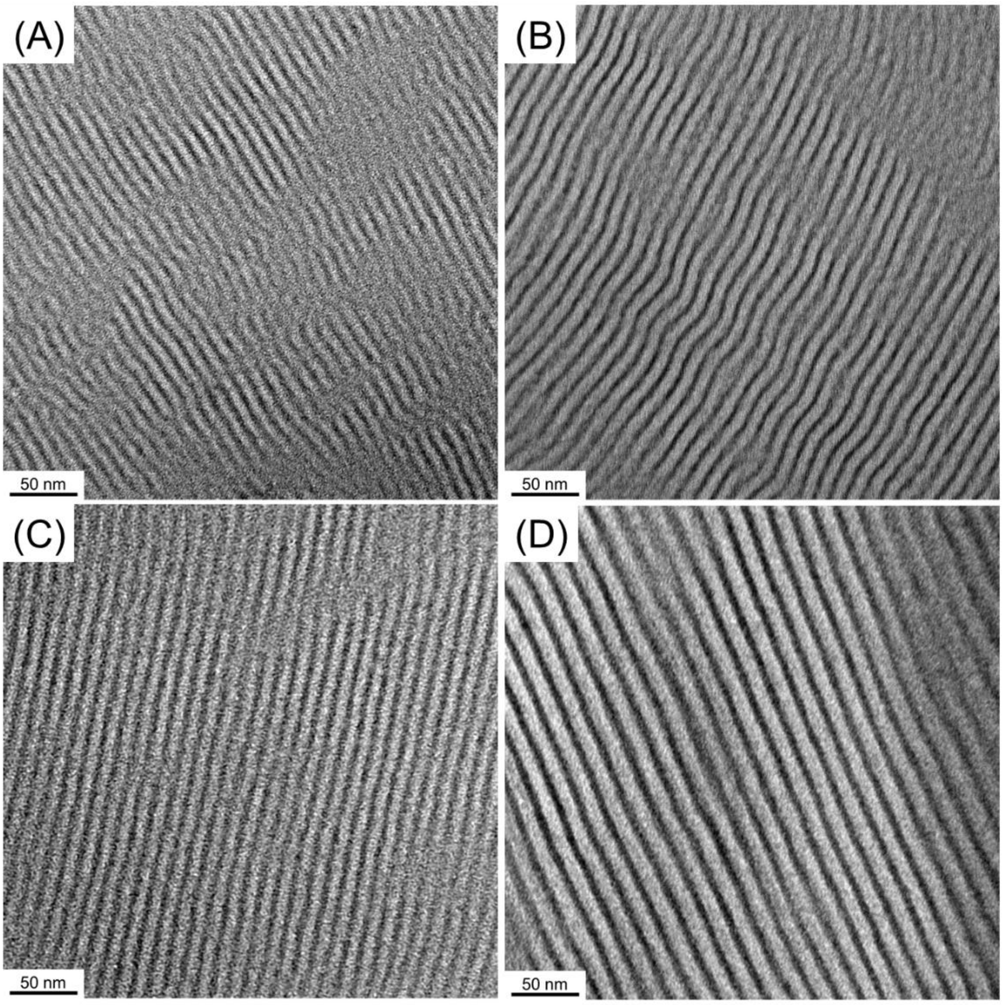

Figure S3. TEM micrographs of the of PS-*b*-PDMS with different molecular weights: (A) $PS_{3.0}$-*b*-$PDMS_{3.5}$, (B) $PS_{5.0}$-*b*-$PDMS_{3.4}$, (C) $PS_{3.9}$-*b*-$PDMS_{4.1}$ and (D) $PS_{5.0}$-*b*-$PDMS_{5.0}$.

***In-situ* Temperature-Resolved Small-Angle X-ray Scattering (SAXS) Experiments.**

The measurements of the temperatures of order-disorder transition (T_{ODT}) obtained from solution casting by cyclohexane were studied by an *in-situ* temperature-resolved SAXS experiments. The measurements are divided into two stages. In the first stage, owing to the gap between the T_{ODT}s and vitrification of PS microdomains below the temperature of glass transition of PS (T_g^{PS}~67□), a rapid heating rate at 20□/min was set till reaching the approximately predicted temperature at T_{ODT} - 60□. Smaller interval between heating steps was set for the precise measurement for the T_{ODT}s. In the second stage, slower heating rate was set for 0.5□/min capture the starting point of ODT. As shown in **Figures 3** and **S4,** the

complete temperature window covers a wide range of 150□ which is expected to be able to examine the ODTs of the high-χ BCPs before the occurrence of degradation. Clear step changes on the inverse intensity of the self-assembled diblocks can be observed; note that the discontinuity of profiles is attributed to the temperature limit of the heater for *in-situ* SAXS experiments. Similar procedures were applied to the three-arm and four-arm star-blocks. As shown in **Figure S5** and **S6**, the uprising jumps to the temperature dependent curves unambiguously mark the occurrence of ODT. Plots of FWHM and inverse intensity were combined for precise determination of the measured T_{ODT}s of the self-assembled (PS-*b*-PDMS)$_n$ (n = 1, 3 or 4) which were summarized in **Figure 7** and **Table 2**.

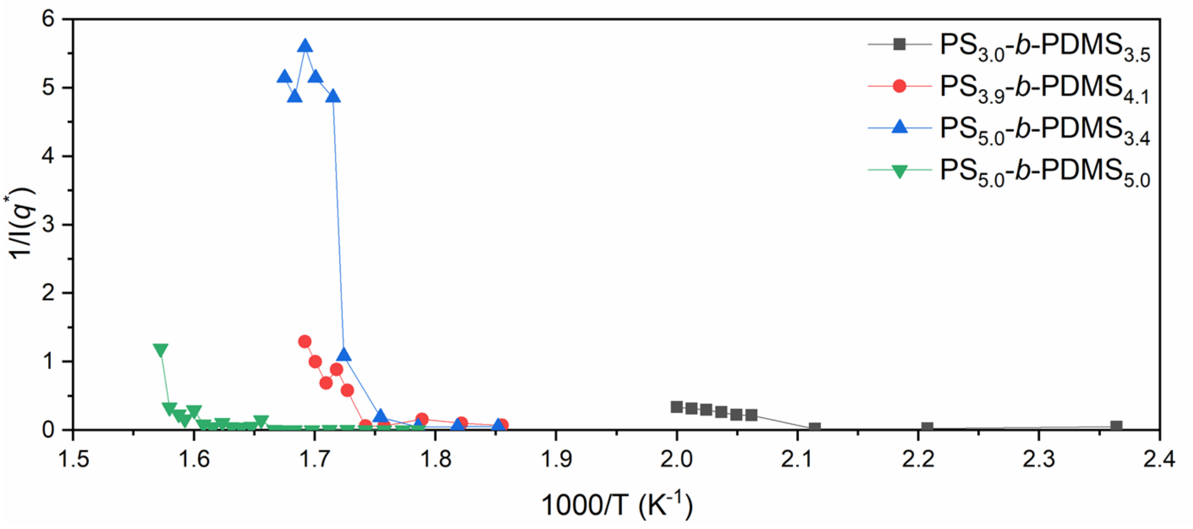

Figure S4. Temperature dependence of inverse of maximum intensity (1/I(q*)) of the self-assembled diblocks derived from the corresponding SAXS profiles shown in **Figures 2A** and **3A-C**.

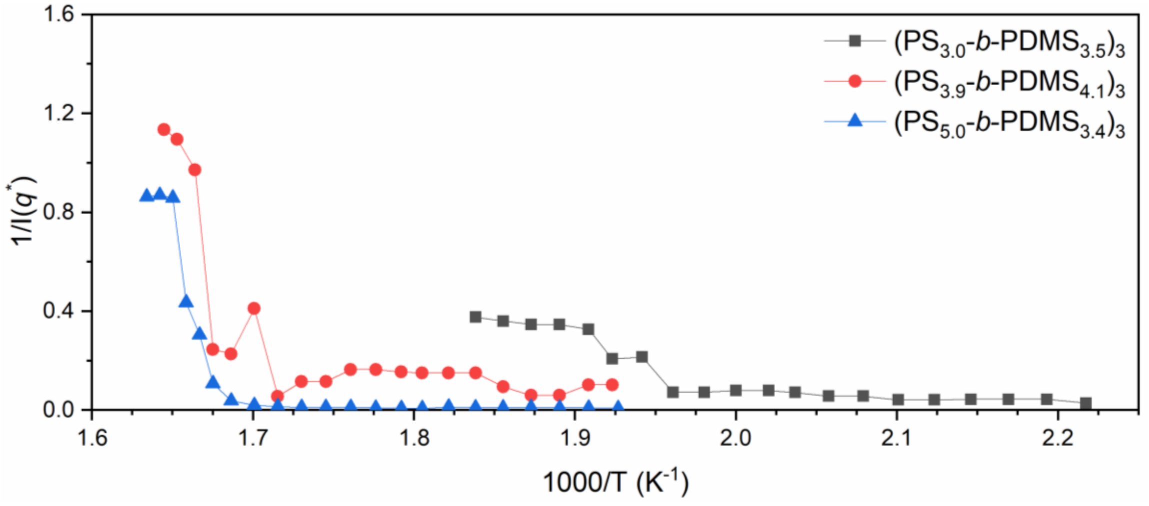

Figure S5. Temperature dependence of inverse of maximum intensity ($1/I(q^*)$) of the self-assembled three-arm star-blocks derived from the corresponding SAXS profiles shown in **Figures 5A-C**.

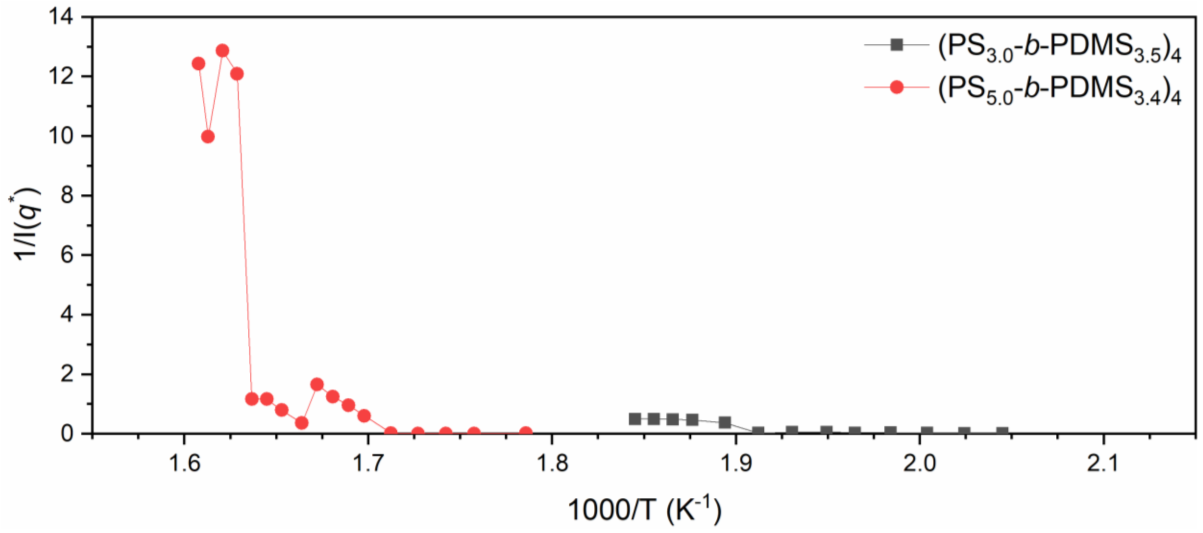

Figure S6. Temperature dependence of inverse of maximum intensity ($1/I(q^*)$) of the self-assembled four-arm star-blocks derived from the corresponding SAXS profiles shown in **Figures 6A** and **B**.

Setups for Prediction of ODTs by Random Phase Approximation (RPA)

In the current study, the calculations of the ODTs for the AB-type diblocks by RPA were carried out following the typical procedures in previous reports.[2-4] For two-component systems, the inverse collective structure factor, $\tilde{S}^{-1}$, was written as follow:

$$\tilde{S}^{\,-1} = \frac{S_{\mathrm{AA}}+2S_{\mathrm{AA}}+S_{\mathrm{BB}}}{2(S_{\mathrm{AA}}S_{\mathrm{BB}}-S_{\mathrm{AB}}^{2})} - \chi,$$

$$\text{where } S_{\alpha\beta} = \frac{1}{\mathrm{N}_t}\int_0^{\mathrm{N}_t} ds_i \int_0^{\mathrm{N}_t} ds_j\, \Theta_{s_i}^{\alpha}\Theta_{s_j}^{\beta} P_{ij},\ \alpha, \beta = \text{A or B}.$$

P_{ij} is the product of the Fourier-space bond transition probabilities of all the segments that form the linear sub-chain bridging segments i and j:

$$P_{ij} = \prod_{\gamma=i}^{j} e^{-\frac{\mathrm{b}_\gamma{}^2 q^2}{6}}$$

with b_γ denoting the statistical segment length of the monomer, γ, and

$$\Theta_{s_i}^{\alpha} = \begin{cases} 1, & \text{if } i\text{th monomer is of type } \alpha \\ 0, & \text{otherwise} \end{cases}$$

The stability limit of the homogeneous phase (*i.e.,* spinodal point) when the ODT occurs is determined by locating the point at which $\tilde{S}$ diverges, or $\tilde{S}^{\,-1}$ goes to zero.

For the incompressible melt of star-blocks, we assume that each AB-type star-block copolymer (BA)$_n$ chain is composed of n identical AB arms joined together through their A (*i.e.,* PDMS) ends, and the B (*i.e.,* PS) ends are free. The total degree of polymerization of one star-block copolymer chain is $\mathrm{N_t} = n(\mathrm{N_A} + \mathrm{N_B})$. With these assumptions, one can carry out the integrals of $S_{\alpha\beta}$ to get the expressions for the various $S_{\alpha\beta}$'s:

$$S_{\mathrm{AA}} = \frac{\mathrm{N_A}^2}{\mathrm{N_A}+\mathrm{N_B}}\frac{-3-2e^{-x_{\mathrm{A}}}(-2+n)+e^{-2x_{\mathrm{A}}}(-1+n)+n+2x_{\mathrm{A}}}{x_{\mathrm{A}}{}^2},$$

$$S_{\mathrm{BB}} = \frac{\mathrm{N_B}^2}{\mathrm{N_A}+\mathrm{N_B}}\frac{(-1+e^{-x_{\mathrm{B}}})[2+e^{-2x_{\mathrm{A}}}(-1+e^{-x_{\mathrm{B}}})(-1+n)]+2x_{\mathrm{B}}}{x_{\mathrm{B}}{}^2},$$

$$S_{\mathrm{AB}} = \frac{\mathrm{N_A}\mathrm{N_B}}{\mathrm{N_A}+\mathrm{N_B}}\frac{e^{-x_{\mathrm{A}}}(1-e^{-x_{\mathrm{A}}})(1-e^{-x_{\mathrm{B}}})(-1+e^{x_{\mathrm{A}}}+n)}{x_{\mathrm{A}}x_{\mathrm{B}}},$$

$$\text{where } x_\alpha = q^2\frac{b_\alpha^2}{6}N_\alpha \quad (\alpha = \text{A or B}).$$

By setting $n = 1$, the equations above reproduce the results for a simple AB diblock copolymer:

$$S_{\mathrm{AA}} = \frac{\mathrm{N_A}^2}{\mathrm{N_t}}g(x_{\mathrm{A}}),\ S_{\mathrm{BB}} = \frac{\mathrm{N_B}^2}{\mathrm{N_t}}g(x_{\mathrm{B}}),$$

$$S_{\mathrm{AB}} = \frac{\mathrm{N_A}\mathrm{N_B}}{\mathrm{N_t}}h(x_{\mathrm{A}})h(x_{\mathrm{B}}),$$

where $g(x) = \frac{2(e^{-x}+x-1)}{x^2}$ is the Debye function and $h(x) = \frac{1-e^{-x}}{x}$.

With the expressions above, we can locate the χ_{spinodal} at which $\min_q \tilde{S}^{-1} = 0$ for a given set of $\{n, \mathrm{N_A}, \mathrm{N_B}, \mathrm{b_A}, \mathrm{b_B}\}$. It is known that the mean-field phase behavior of diblock copolymers modelled as the standard Gaussian chains only depends on the collective parameter $\chi \mathrm{N}\gamma$, where $\mathrm{N}\gamma$ is a reference degree of polymerization that can be chosen arbitrarily. Here, we choose $\mathrm{N}\gamma$ to be the degree of polymerization of one single AB arm of the $(\mathrm{BA})_n$ copolymer, denoted as N. Different choices of $\{\mathrm{N_A}, \mathrm{N_B}\}$ but with the same overall A volume fraction $f_\mathrm{A} = \mathrm{N_A/N}$ will produce the same $(\chi \mathrm{N})_{\mathrm{spinodal}}$. Therefore, we simply choose N = 1 and re-parameterize the polymer chain by $\{n, f_\mathrm{A}, \mathrm{b_A}, \mathrm{b_B}\}$. As an example, the $\tilde{S}^{-1}(q)$ curves with $\{n, f_\mathrm{A}, \mathrm{b_A}, \mathrm{b_B}\}$ = {3, 0.5, 1, 1} and three typical values of χN, *i.e.* 10, 8.14 and 6, are shown in **Figure S7**. In particular, the minimum value of the curve corresponding to χN = 8.14 is 0 and thus is identified as the $(\chi \mathrm{N})_{\mathrm{spinodal}}$ marking at the ODT.

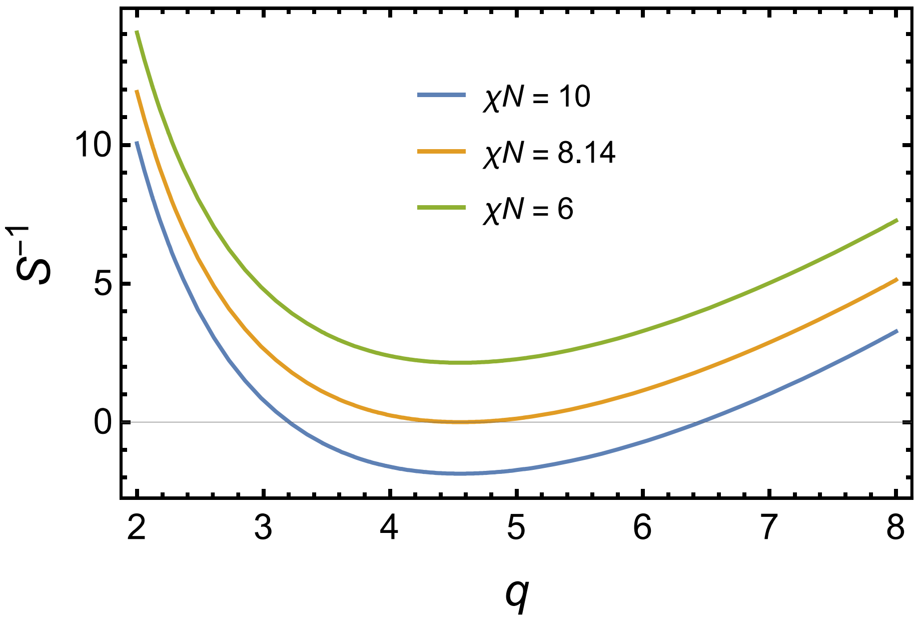

Figure S7. The $\tilde{S}^{-1}(q)$ curves with $\{n, f_\mathrm{A}, \mathrm{b_A}, \mathrm{b_B}\}$ = {3, 0.5, 1, 1} and three typical values of χN, *i.e.* 10, 8.14 ($(\chi \mathrm{N})_{\mathrm{spinodal}}$) and 6.

By using RPA, we evaluate the spinodal curves for the $(BA)_n$ copolymer melts with n ranging from 1 (diblocks) up to 5 ($(BA)_5$ star-blocks), with $b_A = b_B = 1$ chosen for simplicity. The results are presented in the f_A - χN plane and displayed in **Figure S8**. It is clearly seen that the $(\chi N)_{spinodal}$ at the same f_A decreases as increasing n, indicating an increased T_{ODT} as increasing the number of arms of the star-block copolymers.

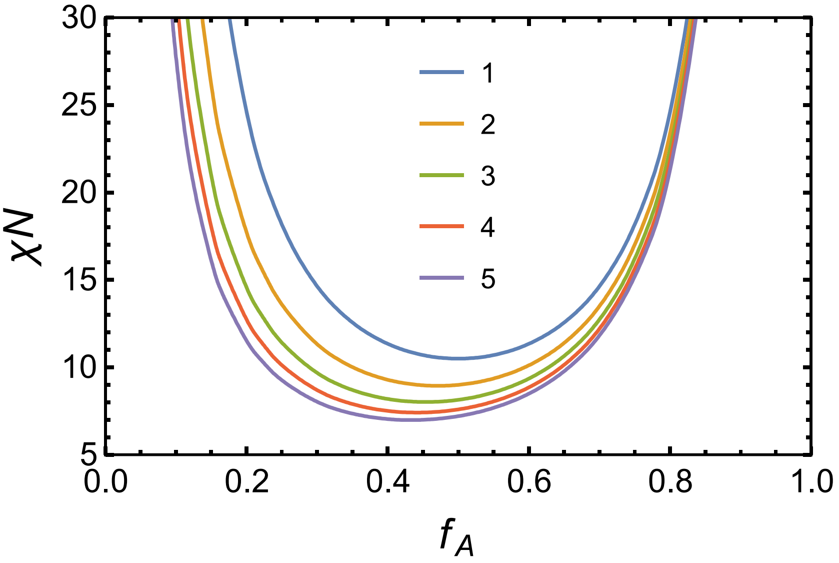

Figure S8. Spinodal curves of the $(BA)_n$ star-block copolymer melts with different n.

Measurements of Latent Heat of Self-Assembled (PS-*b*-PDMS)$_n$ at ODTs

As demonstrated in previous study, the composition fluctuation inhomogeneity at ODTs can be measured by differential scanning calorimetry (DSC).[5] More predictions of the latent heat of diblocks at the ODTs have been also investigated.[6, 7] Here, we aim to study the topology effect on the phase behaviors of the self-assembled high-χ BCPs, especially for the difference on the degree of fluctuation on polymer chains. The heating and cooling rates for scanning of the DSC thermogram are fixed at 20°C/min, ranging from 150°C to temperatures above the measured T_{ODT} by 10°C. Cyclic heating and cooling procedures were adopted for examination of the reversible ODTs. The heating and cooling curves are plotted in separated figures as shown in **Figure S9**. The summary of the measure latent heat of ODTs from the heating and cooling cycles are summarized in **Table S1**.

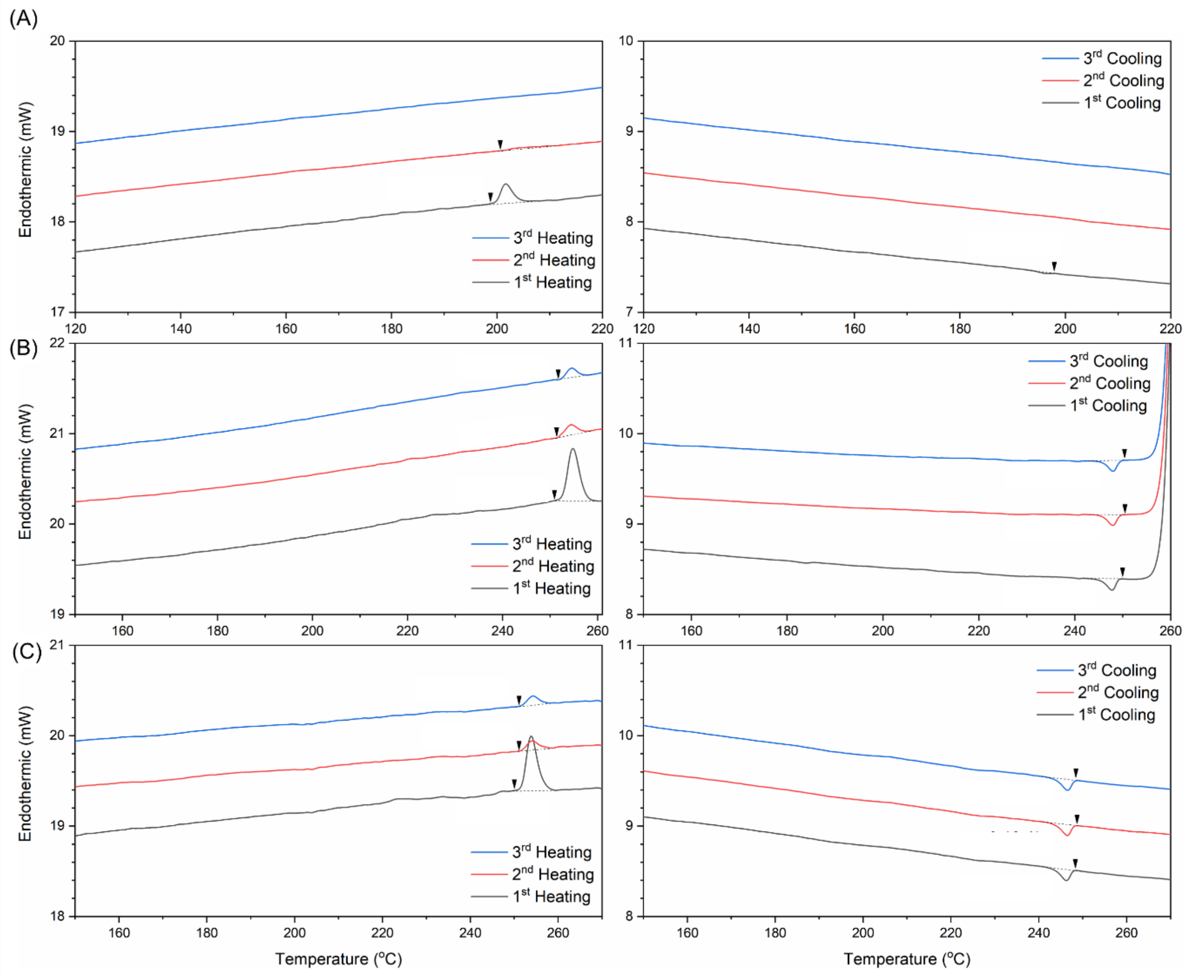

Figure S9. DSC thermograms of the self-assembled (A) $PS_{3.0}$-*b*-$PDMS_{3.5}$, (B) ($PS_{3.0}$-*b*-$PDMS_{3.5}$)$_3$, (C) ($PS_{3.0}$-*b*-$PDMS_{3.5}$)$_4$ after solution casting and followed by thermal annealing. The measurements of the latent heat at ODTs (ΔH_{ODT}) were calculated from the first cycle during measurements at the heating and cooling rates of 20℃/min.

Table S1. Summary of the measured latent heat at ODT during the heating and cooling cycles derived from the DSC thermograms in **Figure S9**. The endothermic heat flow is set as positive value.

	$PS_{3.0}$-b-$PDMS_{3.5}$	$(PS_{3.0}$-b-$PDMS_{3.5})_3$	$(PS_{3.0}$-b-$PDMS_{3.5})_4$
1st heating (ΔH, T)	0.22 J/g, 201°C	0.58 J/g, 252°C	0.68 J/g, 257°C
1st cooling (ΔH, T)	-0.01 J/g, 196°C	-0.10 J/g, 246°C	-0.12 J/g, 248°C
2nd heating (ΔH, T)	0.02 J/g, 203°C	0.14 J/g, 252°C	0.14 J/g, 257°C
2nd cooling (ΔH, T)	-	-0.10 J/g, 246°C	-0.12 J/g, 248°C
3rd heating (ΔH, T)	-	0.11 J/g, 252°C	0.11 J/g, 257°C
3rd cooling (ΔH, T)	-	-0.10 J/g, 246°C	-0.12 J/g, 248°C

References

(1) Georgopanos, P.; Lo, T.-Y.; Ho, R.-M.; Avgeropoulos, A. Synthesis, molecular characterization and self-assembly of (PS-*b*-PDMS)$_n$ type linear (n = 1, 2) and star (n = 3, 4) block copolymers. *Polym. Chem.* **2017**, *8* (5), 843-850.

(2) de Gennes, P. G.; Witten, T. A. Scaling Concepts in Polymer Physics. *Physics Today* **1980**, *33* (6), 51-54.

(3) Leibler, L. Theory of Microphase Separation in Block Copolymers. *Macromolecules* **1980**, *13* (6), 1602-1617.

(4) Whitmore, M. D.; Noolandi, J. Theory of phase equilibria in block copolymer-homopolymer blends. *Macromolecules* **1985**, *18* (12), 2486-2497.

(5) Jun, T.; Lee, Y.; Jo, S.; Ryu, C. Y.; Ryu, D. Y. Composition Fluctuation Inhomogeneity of Symmetric Diblock Copolymers: χN Effects at Order-to-Disorder Transition. *Macromolecules* **2018**, *51* (1), 282-288.

(6) Helfand, E.; Tagami, Y. Theory of the interface between immiscible polymers. II. *J. Chem. Phys.* **1972**, *56* (7), 3592-3601.

(7) Lee, S.; Gillard, T. M.; Bates, F. S. Fluctuations, Order, and Disorder in Short Diblock Copolymers. *AIChE J.* **2013**, *59* (9), 3502-3513.
